# Nonwoven Ion-Exchange Membranes with High Protein Binding Capacity for Bioseparations

**DOI:** 10.3390/membranes11030181

**Published:** 2021-03-06

**Authors:** Solomon Mengistu Lemma, Cristiana Boi, Ruben G. Carbonell

**Affiliations:** 1 Golden LEAF Biomanufacturing Training and Education Center (BTEC), North Carolina State University, 850 Oval Dr, Raleigh, NC 27695-7905, USA; smlemma@ncsu.edu (S.M.L.); rgcarbon@ncsu.edu (R.G.C.); 2 DICAM, Alma Mater Studiorum-Università di Bologna, via Terracini 34, 40128 Bologna, Italy; 3 Department of Chemical and Biomolecular Engineering, North Carolina State University, 911 Partners Way, Raleigh, NC 27695-7905, USA

**Keywords:** nonwoven membrane, UV-grafting, membrane chromatography, ion-exchange membrane, protein purification, membrane adsorbers

## Abstract

This study presents the preparation and characterization of UV-grafted polybutylene terepthalate (PBT) ion exchange nonwoven membranes for chromatographic purification of biomolecules. The PBT nonwoven was functionalized with sulfonate and secondary amine for cation and anion exchange (CEX and AEX), respectively. The anion exchange membrane showed an equilibrium static binding capacity of 1300 mg BSA/g of membrane, while the cationic membranes achieved a maximum equilibrium binding capacity of over 700 mg hIgG/g of membrane. The CEX and AEX membranes resulted in dynamic binding capacities under flow conditions, with a residence time of 0.1 min, of 200 mg hIgG/mL of membrane and 55 mg BSA/mL of membrane, respectively. The selectivity of the PBT-CEX membranes was demonstrated by purifying antibodies and antibody fragments (hIgG and scFv) from CHO cell culture supernatants in a bind-an-elute mode. The purity of the eluted samples exceeded 97%, with good log removal values (LRV) for both host cell proteins (HCPs) and DNA. The PBT-AEX nonwoven membranes exhibited a DNA LRV of 2.6 from hIgG solutions in a flow-through mode with little loss of product. These results indicate that these membranes have significant potential for use in downstream purification of biologics.

## 1. Introduction

The universal prevalence and awareness of various chronic and deadly diseases continue fueling the worldwide market demand for therapeutic proteins, vaccines, and gene vector manufacturing in the biopharmaceutical industry. The global biopharmaceutical market is projected to reach half a trillion dollars by 2025 [1,2]. The production of biologics requires reliable and efficient separation processes to ensure the safety, potency and efficacy of the therapeutic [3,4]. The purification processes used to meet regulatory requirements tend to be expensive and exhibit relatively low productivities [2,5,6]. Studies have reported that more than 80% of the total cost of the manufacturing of biotherapeutics is attributed to the downstream process isolation and purification steps [7,8]. Large scale packed resin column chromatography is the dominant unit operation used for product capture and polishing in the biopharmaceutical industry [9,10,11]. The resins can be costly (up to $20,000 per liter), they require special techniques for packing, they require cleaning and validation after use, and the pressure drop in the column limits the residence time and productivity of the process. Due to this, the column chromatography step, on its own, contributes a significant portion of overall manufacturing costs [10,12].

For these reasons, membrane chromatography has become a promising alternative to columns due to the very low membrane production costs, and their potential to serve as single-use devices [12,13]. Membrane ion-exchange chromatography promises several advantages over column chromatography, including low operating pressure and fast flow rates, ease-of-use, and elimination of cleaning and validation costs in single use applications [14,15,16]. A great deal of work has been done on nonwoven membrane chromatography [6,13,17,18]. The main focus has been on how to make maximum use of the surface area for protein binding, and surface functionalization methods for various uses [6,17,19,20]. The functionalization steps can impact the performance by weakening the fibers, and blocking or occluding the pores to cause an increased pressure drop [6,13]. In addition, the fiber diameter, membrane thickness and porosity, pore size distribution, grafting conditions, and weight gain of the grafted layers are also properties affecting the performance of nonwoven ion-exchange membranes.

For protein purification applications, it is extremely important to have a high binding capacity for the desired proteins with low non-specific adsorption of unwanted host cell proteins. Previous work has studied the grafting of glycidyl methacrylate (GMA) on nonwoven membranes using ultraviolet (UV) light irradiation and heat-induced polymerization, and attaching ligands through the epoxy end groups of the GMA [17,19,21,22]. It is clear from these studies that GMA formed uniform and conformal coatings on the polybutylene terepthalate (PBT) nonwovens [6,17]. The grafted layer thickness, as measured by the % weight gain of the membrane upon grafting plays a key role in membrane performance. GMA grafted layers were successfully functionalized with sulfonate ligands [17,19] to result in cation exchange supports and with diethylamine (DEA) to produce anion exchanger materials [6,17]. The permeability of these materials and their static and dynamic protein binding were measured. Ion exchange membranes have been used in various biologics purification applications both in a bind-and-elute mode for product capture as well as a flow-through mode for impurity removal, or polish [16,20,23,24].

This study was aimed at the optimization of the dynamic binding capacities for protein binding of UV grafted nonwoven membranes modified with ion exchange ligands, and to test their specificity and selectivity in real applications aimed at the purification of proteins from complex mixtures such as Chinese Hamster Ovary (CHO) cell supernatants. The PBT-GMA grafted layers were prepared using UV- irradiation with a range of exposing times to control the % weight gain during grafting, and thus the grafted layer thickness. These UV-grafted membranes were functionalized to produce PBT-nonwoven anion and cation exchange membranes by covalently attached sulfonate (SO_3_) and DEA, respectively. The physical properties of the ion exchange membranes were measured to determine their porosity and flow properties as well as their charged ligand densities and how they affect the anion and cation ion exchange static and dynamic binding capacities for bovine serum albumin (BSA) and human immunoglobulin G (hIgG), respectively. These optimized cationic membranes were employed in the purification of human monoclonal antibodies (hIgG) and single-chain variable fragments (scFv) from Chinese hamster ovary (CHO) cell culture supernatant. In addition, the diethylamine ligand membranes were used to evaluate the removal capacities of DNA from proteins solutions. The results indicate that this type of membrane offers significant potential for use in industrial applications.

## 2. Materials and Methods

### 2.1. Materials

Polybutylene terephthalate (PBT) meltblown nonwovens were kindly provided by Macopharma (Tourcoing, France). The samples possess a basis weight of 55.5 g/m^2^, an average fiber diameter of 3.0 μm ± 0.9 μm, and a 194.74 μm ± 9.05 μm thickness.

Glycidyl methacrylate (GMA), (>97%) was purchased from Reagent World, Inc. (Ontario, CA, USA). Photo-initiator benzophenone (BP), (99%) was purchased from Sigma-Aldrich (St. Louis, MO, USA). Borosilicate 1 mm thick micro-slides glass, methanol (HPLC grade), tetrahydrofuran (THF, HPLC grade), 1-butanol (ACS grade), diethylamine (DEA, reagent grade), sodium sulfate (99.5%), isopropanol (>99.5%), sodium acetate trihydrate, tris(hydroxymethyl)aminomethane (Tris base, ≥99%) and sodium chloride (NaCl), (>99%) were all purchased from Fisher Scientific (Fairlawn, NJ, USA). Sodium sulfite (>98+%), hydrochloric acid, and sulfuric acid (95–98%) were purchased from Acros (Morris Plains, NJ, USA). Bovine Serum Albumin (BSA) (≥96%, free of IgG) was purchased from Sigma Aldrich (St. Louis, MO, USA). Human Plasma Immunoglobulin G (hIgG) (99.9% purity) was purchased from Athens Research & Technology, Inc. (Athens, GA, USA). CHO HCP ELISA Kit (F550) and CHO DNA concentrate (D552) were purchased from Cygnus (Southport, NC, USA) and Quant-iT™ PicoGreen™ dsDNA Assay Kit was from Fisher Scientific, Fairlawn, NJ, USA. Sodium dodecyl sulphate-polyacrylamide gel electrophoresis (SDS-PAGE) kits were purchased from Bio-Rad (Hercules, CA, USA). All kits were used according to manufacturer’s instructions. Chinese Hamster Ovary (CHO) cell supernatant and single-chain variable fragment (scFv) (98.5% purity) were obtained from the Golden Leaf Biomanufacturing Training and Education Center (BTEC) at NC State University.

Buffers were prepared from analytical-grade chemicals and deionized water (Milli-Q system fitted with BioPak^®^ polisher, MilliporeSigma, Bedford, MA, USA). 20 mM Sodium acetate buffer, pH 5.5 (for cation exchange membranes), and 20 mM Tris-HCl buffer, pH 7.0 (for anion exchange membranes) were used as equilibration buffers in protein binding experiments. Elution buffers were prepared by adding 1 M NaCl to the respective equilibration buffers and adjusting the pH values with 100 mM NaOH and HCl.

### 2.2. Membrane UV-Grafting with GMA

The PBT nonwoven membrane was weighed before grafting. UV grafting with GMA was performed at different GMA concentrations and grafting times to obtain optimized conditions. The GMA received without inhibitor was used to prepare a (20%, *v*/*v*) GMA grafting solution with 1-butanol as the solvent. BP was added to the grafting solution as a photo-initiator with a molar ratio BP:GMA of 1:20 as previously reported [17]. Weighed nonwoven PBT samples (75 × 50 mm) were wetted by dripping with 1 mL GMA grafting solution evenly over the membrane surface. The samples were then sandwiched between two borosilicate glass slides and immediately UV irradiated (lamp model EN-180, Spectronics Corporation, Westbury, NY, USA) at a wavelength of 365 nm and 5 mW/cm^2^ intensity to polymerize the GMA on the membranes. The distance between the nonwoven samples and the lamp was 3 mm. The samples were irradiated at various exposure times to achieve different degrees of polyGMA grafting with different % weight gains.

After grafting, the membranes were soaked in 25 mL THF per membrane sample and sonicated in an ultrasonic bath (CPX962118R, Thermo Fisher Scientific, Pittsburgh, PA, USA) for 30 min at room temperature to remove unreacted GMA and benzophenone. The samples were subsequently sonicated in 25 mL fresh methanol for 10 min to remove THF, followed by drying overnight at room temperature. The nonwoven membranes were weighed to determine the degree of GMA grafting (percentage weight gain), which was calculated as follows:

(1) $$\mathrm{Degree}\;\mathrm{of}\;\mathrm{GMA}\;\mathrm{grafting}\;\%\;=\;\frac{w_{G}-w_{0}}{w_{0}}\;\times 100$$

where *w*_0_ is the initial weight of PBT-nonwoven before grafting and *w_G_* is the weight of GMA grafted membrane [13].

### 2.3. Preparation of Ion-Exchanger Nonwoven Membranes

To create anion exchange membranes, polymerized GMA-grafted membranes (PBT-GMA) were loaded into a 50% (*v*/*v*) DEA aqueous solution at 30 °C under agitation at 100 rpm. The ring-opening reaction between epoxy and DEA was used to covalently attach amino groups on the surface of the fibers. Following amination, the membranes were washed with 100 mL deionized (DI) water to remove unreacted DEA [6].

**Figure 1:**
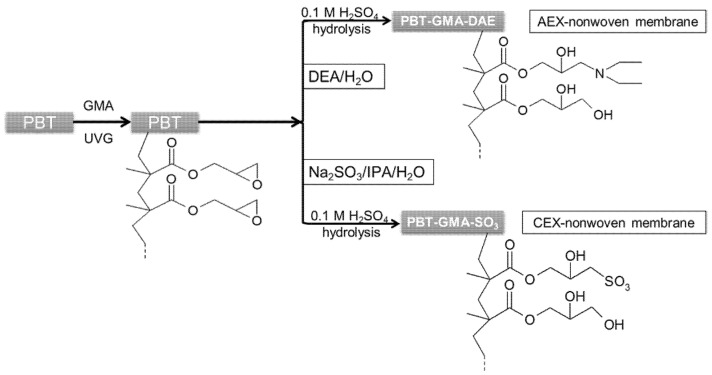
Schematic representation of ion-exchange PBT nonwoven membrane preparation chemistry.

Similarly, to create cation exchange membranes, PBT-GMA samples were immersed into 50 g of a sodium sulfite solution containing isopropyl alcohol (IPA), and water (Na_2_SO_3_/IPA/Water, 10/15/75%, *w*/*w*) at 80 °C without shaking. For both anion (PBT-GMA-DEA) and cation (PBT-GMA-SO_3_) exchangers, the functionalizations were performed at different reaction times and at different ligand densities by varying the initial concentration of DEA and sodium sulfite in the reaction mixtures. After functionalization, cation and anion exchange membranes were hydrolyzed in 0.1 M sulfuric acid solution at 50 °C for 16 h to reduce the remaining epoxy groups as to avoid nonspecific protein binding [21]. Finally, the membranes were thoroughly washed with an excess of Milli-Q water until the pH reached 7.0 and dried overnight at room temperature. A schematic of the chemistry used for UV-grafting and immobilization of ion-exchange ligands on the PBT nonwoven membranes is shown in Figure 1. The functionalized membranes were stored either at 4 °C or at room temperature in sealed plastic bags until use.

### 2.4. Characterization of Grafted Membranes

#### 2.4.1. Morphology

The surface morphology of the grafted and non-grafted samples were observed using a field-emission scanning electron microscope (FEI Verios 460L, Hillsboro, OR, USA) operating at 2 to 30 kV. Prior to analysis, the membrane samples were coated with a thin layer of Au/Pd alloy for five minutes to increase the resolution of the image. The average fiber diameter (AFD) for each sample was calculated from fiber diameters measured for 100 randomly selected fibers from each SEM image.

#### 2.4.2. Thickness and Porosity

The thicknesses of the functionalized membrane samples were measured by a micrometer (AMES 99.0697Q, Framingham, MA, USA with a 0.001 mm accuracy); measurements were taken at different positions on the membrane surface to test their homogeneity. The porosity (*ϕ*) of the nonwoven membranes was calculated using the equation:

(2) $$\phi \;=\;1-\frac{\rho_{m}}{\rho_{b}}$$

where, $\rho_{m}$ is the apparent density of the nonwoven membranes, calculated by weighting a unit area (cm^2^) and measuring the average thickness of each sample, and $\rho_{b}$ is the PBT bulk density, equal to 1.31 g/cm^3^ at 25 °C. The porosities of the nonwoven membranes were calculated before and after functionalization.

#### 2.4.3. Determination of Ligand Density

Ligand densities of the functionalized membranes were determined through elemental analysis. The nitrogen content of the DEA functionalized membranes was analyzed with a PE 2400 CHN elemental analyzer (Perkin Elmer Inc., Waltham, MA, USA) by combusting to gases CO_2_, H_2_O, and N_2_. The total nitrogen was used to quantify the ligand density of the anion exchange membranes. The sulfur content of sulfonated membrane was measured by Ion-Coupled Plasma Spectrometer (ICP-OES 8000, Perkin Elmer Inc., Waltham, MA, USA). The total available sulfur reading of the samples was utilized to determine the ligand density of the cation exchange membranes.

#### 2.4.4. Permeability Measurements

Pressure drops were measured on a membrane stack of 12 layers, total bed height of about 0.30 cm, packed into an adjustable Omnifit EZ Glass column fitted with 30 µm frits (2.5 cm internal diameter, Cambridge, UK). The packed column was connected to a fast protein liquid chromatography, FPLC system (ÄKTA Avant-150, GE Healthcare Bioscience, Uppsala, Sweden), and the column pressure drops were recorded using the internal pressure measurement device present in the feed delivery system of the FPLC. The experiments were performed by feeding Milli-Q water, 20 mM acetate buffer, pH 5.5 (for CEX- membranes), and 20 mM Tris-HCl buffer, pH 7.0 (for AEX-membranes) at different superficial velocities, from 1.22 to 917 cm/h, on the following membrane stacks: native non-grafted nonwoven PBT, UV-grafted (PBT-GMA), PBT-GMA-DEA and PBT-GMA-SO_3_. The Milli-Q water and the buffers were filtered with 0.2 μm pore size sterilized polyethersulfone Thermo Scientific Nalgene filter and degassed by ultrasonication before use.

Prior to the tests, the hydrophobic non-grafted membranes were immersed into 20%, *v*/*v* ethanol aqueous solutions for 30 min to completely wet the porous material. All experiments were performed in triplicate and the data were averaged.

### 2.5. Determination of the Membrane Binding Capacity and Selectivity

Binding experiments were performed in competitive and non-competitive conditions on both anion and cation exchange membranes. In all cases, the equilibration and binding buffer was 20 mM Tris-HCl buffer pH 7.0 for anion exchangers, and 20 mM acetate buffer pH 5.5 for cation exchangers. The elution buffers were prepared by adding 1 M NaCl to the relevant equilibration buffer and correcting the pH to the initial value. The equilibrating buffers preparation is briefly described in the Materials section.

#### 2.5.1. Static Equilibrium Binding Capacity

Equilibrium binding capacity was measured by incubating overnight 10 mg of membrane samples in 3 mL protein solution in a fritted SPE tube, at 10 mg/mL concentration. BSA was chosen as a model protein for anion exchangers, while polyclonal hIgG was used for cation exchangers. Before the experiments, the membranes were thoroughly washed five times in the relevant equilibration buffer. After binding overnight, the membranes were washed five times with the equilibration buffer to remove all the unbound proteins. The bound proteins were recovered by incubating the membranes for two hours in the elution buffer, since a 1 M NaCl concentration in the elution buffer successfully disrupts the ionic interactions to remove all the bound proteins from the charged nonwoven membranes [17]. Static binding experiments were performed at room temperature under mild agitation. The eluted protein concentration was measured through absorbance readings at 280 nm using UV-vis spectroscopy (Agilent Technologies, G1103A, Santa Clara, CA, USA). All measurements were done in triplicate and average values were reported to determine the amount of protein adsorbed by the membranes.

#### 2.5.2. Flow Experiments—Dynamic Binding Capacity

Dynamic binding experiments were performed using the same set-up as the water permeability tests described above. The membrane column of 12 layered stacks measured 1.5 mL bed volume corresponding to approximately 0.45 g of dried membrane weight. The dry membranes were wetted by immersing them in equilibration buffer for 10 min before packing the column. The buffers were simultaneously degassed and pre-filtered through a 0.2 µm PES sterile membrane (Thermo Scientific™ Nalgene) using a vacuum pump. The flow rate was set to 1.0 mL/min (12.2 cm/h) for equilibration, washing, and elution, while the loading stage was performed at different flow rates, from 0.1 to 15 mL/min, corresponding to 10 to 0.1 min residence time (RT) depending on the type of experiment.

Pure protein solutions were used in non-competitive conditions to measure the dynamic binding capacity at saturation, i.e., 100% breakthrough. To this aim, BSA was used as a model protein for anion exchange PBT-GMA-DEA membranes and polyclonal hIgG for cation exchange PBT-GMA-SO_3_ membranes. Protein solutions in equilibration buffers were tested in a concentration range from 2 to 10 g/L. The membranes were loaded at flow rates from 0.33 to 15 mL/min (corresponding to velocities of 4 to 183 cm/h and 5 to 0.1 min RT) until 100% breakthrough to determine the dynamic binding capacity. Moreover, complete bind and elute tests at 0.5 min RT were performed over six cycles to investigate the ion-exchange membrane binding performance for reusability. The dynamic binding capacity was calculated by measuring the total amount of protein recovered in the eluted fractions divided by the column volume or by the dry membrane weight.

The membranes were tested in competitive conditions using CHO supernatants, in particular, cation exchange membranes were challenged in bind-and-elute mode with spiked polyclonal hIgG or scFv. The feed sample was prepared by adding a known amount of hIgG or scFv into clarified CHO cell culture supernatant that was diafiltered against the loading buffer (20 mM acetate buffer, pH 5.5), using Macrosep^®^ (3000 MWCO Pall Corporation, NY, USA) to adjust the conductivity and the pH, to have a final titer of 2 mg/mL. Feed samples of 150 mL were loaded at different residence times to completely saturate the column. The unbound proteins were washed with equilibration buffer and the target eluted with 1 M NaCl in equilibration buffer. The chromatographic fractions were collected and analyzed to determine binding capacity, purity of the target protein, and level of impurities removal.

Anion exchange membrane adsorbers are currently used in polishing steps for the removal of impurities. To this aim, DNA was spiked to the two separate proteins, polyclonal hIgG or scFv solutions, in CHO supernatants. The final concentration of DNA was 1 µg/mL in each of the hIgG or scFv solutions at concentrations of 2 mg/mL of protein. The hIgG and scFv solutions were prepared separately with equilibration buffer. The DNA in the protein solution was used as a known amount of DNA impurity in the feed samples. After equilibration, PBT-GMA-DEA membrane columns were loaded with 10 mL of samples at three linear flow velocities of 1.8, 3.7, and 9.2 cm/h (equivalent to 10, 5, and 2 min RT). The DNA bound on membranes was eluted for quantification purposes, using 1 M of NaCl in equilibration buffer. A chromatography detection wavelength of 280 nm was used throughout the experiments. The total amount of DNA in the collected fractions was measured by Picogreen assay to determine the log reduction value (LRV). The fractions were further analyzed by Protein G, SEC-HPLC, SDS-PAGE for purity, and the recovery of the antibodies in the flow-through was calculated.

### 2.6. Analytical Methods

#### 2.6.1. Host cell Protein and DNA Levels Determination

The host cell protein (HCP) content in the CHO supernatant samples were determined using an enzyme-linked immunosorbent assay (ELISA), the 3rd Generation CHO HCP kit (Catalog F550) from Cygnus Technologies (Southport, NC, USA). The DNA levels in the fractions were quantified by Picogreen assay using Quant-iT PicoGreen dsDNA assay kit from Thermo Fisher Scientific (Fairlawn, NJ, USA). The log reduction values (LRV) of HCP and DNA clearance were expressed by log_10_ ratio of HCP or DNA in the load to the elution fractions. In both cases, the analyses were performed by following the manufacturer’s instructions and protocols.

#### 2.6.2. SEC-HPLC Analysis of Collected Fractions

The separation of the scFv in the chromatographic fractions was performed by HPLC using a Size Exclusion Column (SEC) Yarra SEC-2000 column (3 μm, 300 mm × 7.8 mm ID) from Phenomenex (Torrance, CA, USA). The column was equilibrated with PBS at pH 7.4, the mobile phase buffer, at a flow rate of 0.5 mL/min. The injection volume of the samples was 50 µL, the analysis was monitored at 280 nm within 30 min of retention time. The purity of scFv was determined based on the curve area relative to the standard solution.

#### 2.6.3. Protein G Chromatography

Protein G (HiTrap, 5 × 1 mL) affinity resin from Cytiva (Marlborough, MA, USA) was employed to determine hIgG purity in the collected fractions using Fast Protein Liquid Chromatography (FPLC). The hIgG purifications were carried out using the optimized manufacturer’s protocol. Briefly, the column was equilibrated and washed with PBS at pH 7.4. The fractions collected samples (100 µL) were loaded at a flow rate of 86 cm/h (1 mL/min). The samples were eluted with 0.1 mM glycine-HCl at pH 2.7. The UV detection was monitored at 280 nm. Pure hIgG was used as a standard for the peak area estimation. Determination of hIgG purity (%) in the samples was based on the evaluation of the peak area as compared with the standard.

#### 2.6.4. SDS-PAGE and Total Protein

Sodium dodecyl sulphate-polyacrylamide gel electrophoresis (SDS-PAGE) was conducted on 4–15% Mini-PROTEAN^®^ TGX™ Precast Protein Gels from Bio-Rad. The collected fractions were diluted in the 2× Laemmli (Bio-Rad) sample loading buffer (1:1). The samples were reduced by mixing 5% mercaptoethanol and heated at 100 °C for 5 min. Gels loaded with 5 µg total protein per lane were stained with Coomassie-blue for estimating the purity of the proteins and silver-stained gel with 3 µg total proteins of CHO-cell culture supernatant. The total protein content of the samples was determined using the BCA assay kit from Pierce (Rockford, IL, USA).

## 3. Results and Discussion

### 3.1. Surface Modification of Nonwoven Membranes

Prior work has described the UV- and heat-induced grafting processes for creating ion exchange membranes from nonwoven fabrics [6,13,19,21,25]. This study presents recent progress aimed at optimizing the dynamic binding capacities of these membranes under flow conditions at a wide range of residence times, and testing the ability of the membranes to purify proteins from real cell culture supernatants. The focus is on membranes prepared with higher grafting densities than those used in prior efforts. New results are presented on the optimization of ligand densities (Figure S1), and on the membrane stability during storage (Figure S2). This work also describes in more detail the pressure drop characteristics of the membranes, which play a crucial role in enabling large superficial velocities and short residence times. 

**Figure 2:**
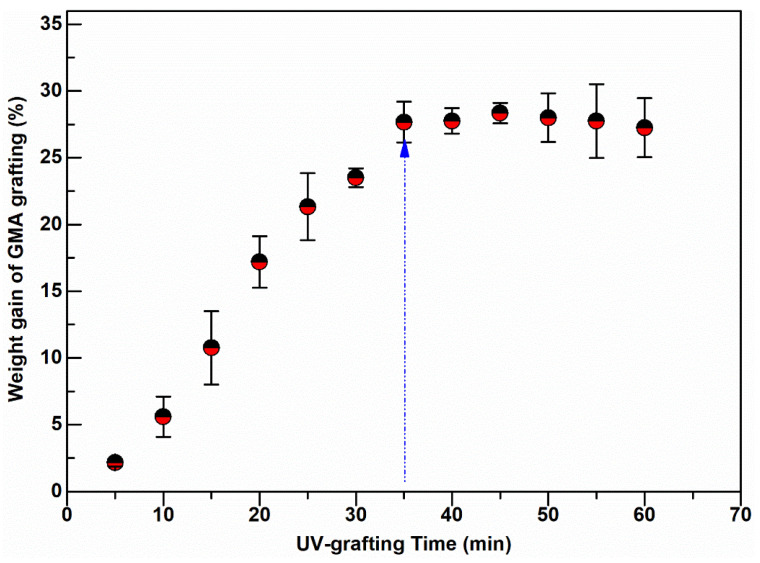
Weight gain of UV-grafted PBT nonwoven membranes at various exposure times.

Surface grafting on nonwoven membrane fibers was carried out at various degrees of GMA polymerization induced by UV-light radiation using different exposure times, between 5 and 60 min. The grafting degree, measured in terms of % weight gain of the nonwoven due to grafting, has an initial linear growth region and a short plateau, followed by a slight decline in weight gain as shown in Figure 2. The weight gain increased with the UV exposure time up to 35 min, when the grafting reached a plateau.

**Figure 3:**
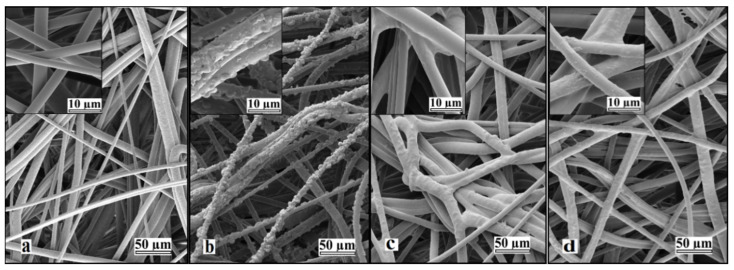
Representative SEM images of nonwoven PBT membranes: (**a**) non-grafted PBT, (**b**) UV-grafted PBT-GMA (20% weight gain), (**c**) cation exchanger PBT-GMA-SO_3_, and (**d**) anion exchanger PBT-GMA-DEA. The insets show higher magnification images.

Prolonged UV irradiation after 45 min resulted in degradation of the material and a loss in measured weight gain. This degradation can be observed in Figure 2. Similar UV-grafting regions were reported on cellulose fabrics modified using methyl methacrylate (MMA) and polyacrylamide in the presence of benzophenone at different grafting times [26,27,28]. The morphological changes induced on the grafted PBT nonwoven membranes were observed by SEM as shown in Figure 3. The images confirmed that the UV-grafted PBT-GMA, Figure 3b, has a rough surface and generally larger fiber diameters as compared with blank PBT. However, the DEA and SO_3_ functionalized layers exhibited uniform and smooth fibers and resulted to an increase of the average fiber diameter of about 12% with respect to the blank PBT, as shown in Figure 3c,d. This increase in fiber diameter, determined from image analyses, indicates a clear presence of grafted layers on the surface of the fibers while the pores between the fibers in the original nonwoven are maintained. In the studies described below, only 20% weight gain membranes were used. In a previous study in our lab, low degrees of GMA grafting (<10% weight gain) did not achieve complete fiber coverage required to render the membrane surface hydrophilic [25]. On the contrary, PBT nonwoven membranes with weight gains in the range of 10 to 30%, exhibited more uniform, conformal grafted layers and excellent membrane physical integrity as compared with surfaces having higher and lower weight gain. In addition, the membranes exhibit complete uniform GMA grafted coverage as shown in the SEM images of the fibers before (Figure 3a) and after (Figure 3b) grafting at 20% weight gain.

### 3.2. Membrane Porosity and Permeability

#### 3.2.1. Porosity and Morphological Changes

Unmodified PBT nonwoven membranes are endowed with high porosity, 79.6%, prior to any treatment. The porosity of the nonwovens decreased slightly upon surface modification. The porosity of PBT-GMA, of the cation exchanger (PBT-GMA-SO_3_), and the anion exchanger (PBT-GMA-DEA) decreased to 78.7%, 77.3%, and 76.1%, respectively.

This is consistent with the increased basis weight of grafted and functionalized membranes that present larger fiber diameters as shown in Figure 3. Indeed, this slight porosity reduction is largely compensated by the great availability of surface functionalities that is paramount for bioseparations.

#### 3.2.2. Permeability of Nonwoven Membranes

**Figure 4:**
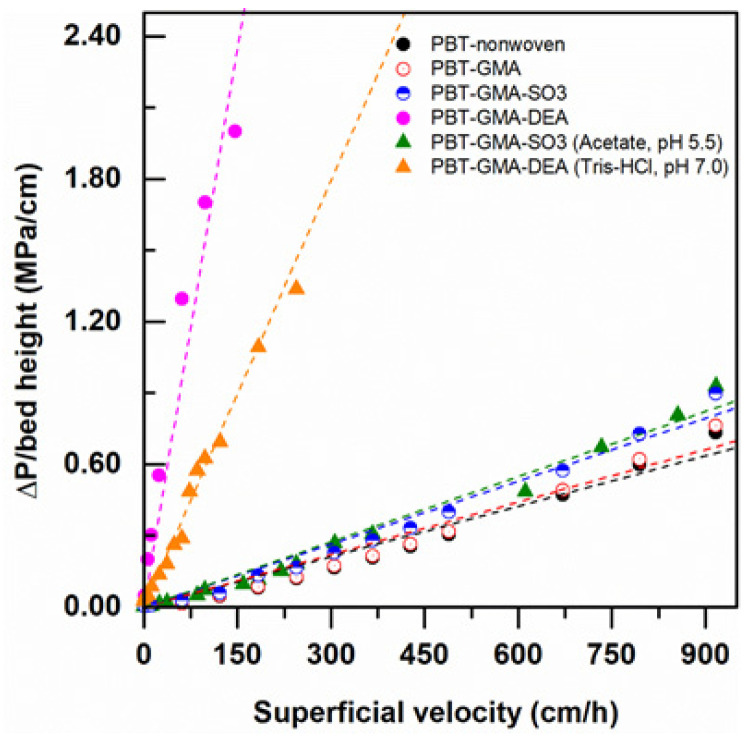
Water and 20 mM buffer pressure drop normalized with membrane bed height versus superficial flow velocity in a 2.5 cm diameter column packed with 12 layers of nonwoven membranes at a bed height of 0.30 cm.

Pressure drop measurements at various superficial velocities were recorded using modified and non-modified nonwoven membranes by feeding Milli-Q water and low ionic strength (20 mM) buffers at various flow velocities, up to 917 cm/h (equivalent to a flow rate of 75 L/min). As shown in Figure 4, the DEA functionalized AEX membranes exhibited higher pressure drops than the CEX membranes with both pure water and in low ionic strength buffer. As will be discussed in more detail below, the AEX membranes have charged ligand densities that are significantly greater than the ligand densities in the CEX membranes. In water, the charged layers can experience significant repulsive forces between charges that induce expansion of the grafts, closure of the pores between fibers, and increased pressure drops. This pore closure effect is much stronger in the higher ligand density AEX membranes than the CEX membranes. 

Comparing the pressure drop data in pure water to the pressure drop data taken in low ionic strength buffer for the DEA membranes, it is clear that the pore closure effect is weaker in the increased ionic strength buffer than in pure water and the observed pressure drops are lower. This is to be expected since the repulsive forces become weaker in the increased ionic strength environment. This effect is not as evident in the lower ligand density CEX membranes, where the pressure drops in pure water and in low ionic strength buffer are very similar. This decrease in swelling of ion exchange membranes and monoliths with increasing charge density and ionic strength has been reported previously in the literature [29,30,31,32].

**Figure 5:**
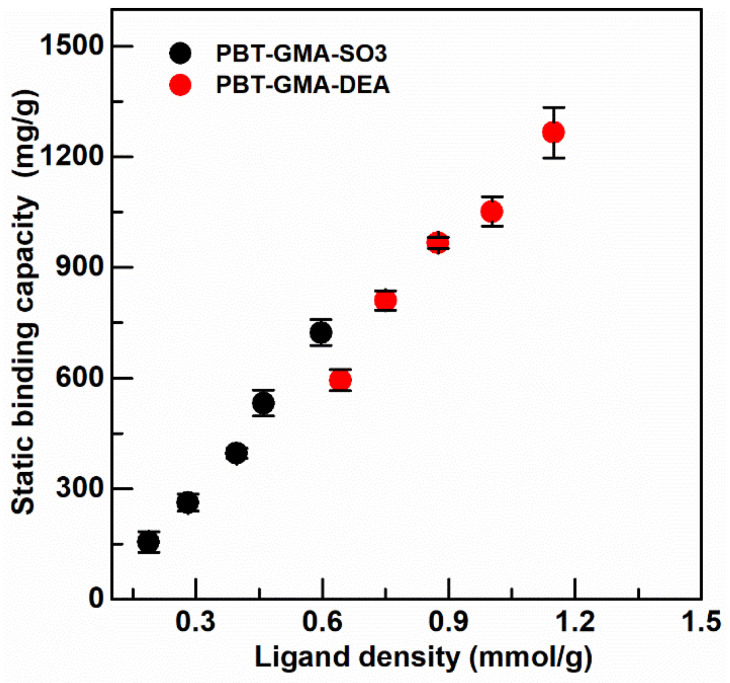
Static binding capacity of BSA on anion exchanger PBT-GMA-DEA, and of hIgG on cation exchanger PBT-GMA-SO_3_ membranes at different ligand densities.

To measure the flow permeability of the membrane column under investigation, Darcy’s law was applied, since even at the highest value of flow rate employed in the experiments, the maximum particle Reynolds number is approximately 0.05. This value was calculated using a water density of 1000 kg m^−3^, a viscosity of 10^−3^ Pa s, and an average pore diameter of 10 µm. Under this viscous flow regime, the pressure drop in porous media follows Darcy’s law [33]

(3) $$v\;=\;\frac{\Delta P}{µL}\kappa$$

where v is the superficial velocity, Δ*P* is the pressure drop along the column of thickness *L*, µ is the viscosity of the fluid, and κ is the flow permeability of the porous medium. The permeability of the nonwoven membranes was determined by linear fitting of the data reported in terms of superficial velocity versus Δ*P*/µ*L* and the results are reported in Table 1, together with the values of porosity and basis weight (membrane mass per unit area). As expected, porosity and permeability decreased with functionalization, whilst the basis weight increased. However, a different behavior is observed for anion and cation exchangers: PBT-GMA-DEA shows a much lower permeability than PBT-GMA-SO_3_ that is not justified solely by porosity reduction. Indeed, the increase in basis weight due to a longer functionalization time and therefore to a much higher ligand density, as is shown in Table 1, is one of the possible explanations. The high ligand density can result in high pressure drops in low conductivity solvents, such as the pure water used in the experiments shown in Figure 5. The membranes work well under a buffer conductivity ~3.0 mS/cm at a flow rate below 20 mL/min, which is more than four times higher than the normal flow rate used in membrane chromatography [8,34].

The values of permeability obtained for the functionalized nonwoven membranes are of the same order of magnitude as the permeability of CIM monolithic media, Trilisky et al. reported a κ value of 6.3 × 10^−15^ m^2^ for CIM DEAE disks [35] that is consistent with a *κ* of 5.74 × 10^−15^ m^2^ obtained by Herigstadt et al. [36] for CIM Protein A disks.

### 3.3. Static Binding Capacity

The static binding capacities of functionalized membranes were measured in batch using two model proteins, polyclonal hIgG for cation, and BSA for anion exchange membranes. A high protein concentration of 10 mg/mL and sufficient time to reach equilibrium, 16 h, were used to ensure that the maximum binding capacity was attained. Control experiments to determine non-specific protein binding were performed with native PBT nonwovens and PBT-GMA membranes. In both cases, the membranes show low levels of non-specific binding, between 6 and 10 mg protein/g of membrane, as is reported in Table 2.

The static binding capacity was measured on ion-exchange membranes, PBT-GMA-DEA, and PBT-GMA-SO_3_, at different values of ligand density (optimized earlier as in Figure S1) to obtain the optimal capacity as shown in Figure 5.

The maximum binding capacity was 1265.53 ± 68.35 mg BSA/g membrane, corresponding to 19.0 ± 1.0 µmol BSA/g membrane, for anion exchange membranes and 712.85 ± 35.11 mg hIgG/g membranes, corresponding to 4.75 ± 0.23 µmol hIgG/g membrane, for cation exchangers. As can be observed in Figure 5, the binding capacity depends significantly on the available functional ligands and surface chemistry of the grafted membranes: an increase in ligand density of ion-exchange groups on the membrane structure corresponds to a high protein adsorption capacity. Previous results with the DEA anion exchange membrane showed a lower static binding capacity (840 mg BSA/g) due to the lower ligand density (0.84 mmol/g) of this membrane [6]. The static binding capacity of the PBT-GMA-DEA membrane is more than two times higher than that of Sartobind-D cellulose membrane [37] and more than four times higher than that of CIM monoliths [38]. However, the static binding capacity of PBT-GMA-SO_3_ for IgG is only three times higher than that of CIM disks, due to the fact that the binding capacity of monoliths increases for higher molecular weight targets [38]. 

It also needs to be mentioned that the static binding capacities of the PBT nonwoven membranes are much greater than those expected when the proteins adsorbed to the fibers in the form of a monolayer. The surface area of the original nonwoven is approximately 1 m^2^/g. With a monolayer of protein adsorbed, this would result in an equilibrium binding capacity of 3–6 mg/g of nonwoven, a value close to those seen in Table 2 for binding capacities to the native nonwoven and the GMA grafted nonwovens. It can only be concluded that the observed binding capacities result from BSA and hIgG diffusing through the grafted layers. Given the large amount of time allowed to reach equilibrium, the proteins permeate through the entire grafted layer. This is not the case in flow experiments carried out to determine the dynamic binding capacities, as described below.

### 3.4. Dynamic Binding Capacity

Membrane chromatography is exceptionally attractive for rapid separations due to the dominant convective transport of proteins to the binding sites [39,40]. The PBT-membrane adsorbers developed in this work were used in chromatographic bind-and-elute cycles to measure the dynamic binding capacity (DBC) at varying residence times. The ligand densities of ion-exchange membranes that offered the highest protein adsorption (Figure 5) were selected for DBC, specifically, PBT-GMA-DEA (~1.4 mmol/g) and PBT-GMA-SO_3_ (~0.40 mmol/g) were used. The experiments, performed with 12 layers of membranes, were conducted at complete saturation to assess the maximum adsorption capacity at different feed concentrations and superficial velocities. 

**Figure 6:**
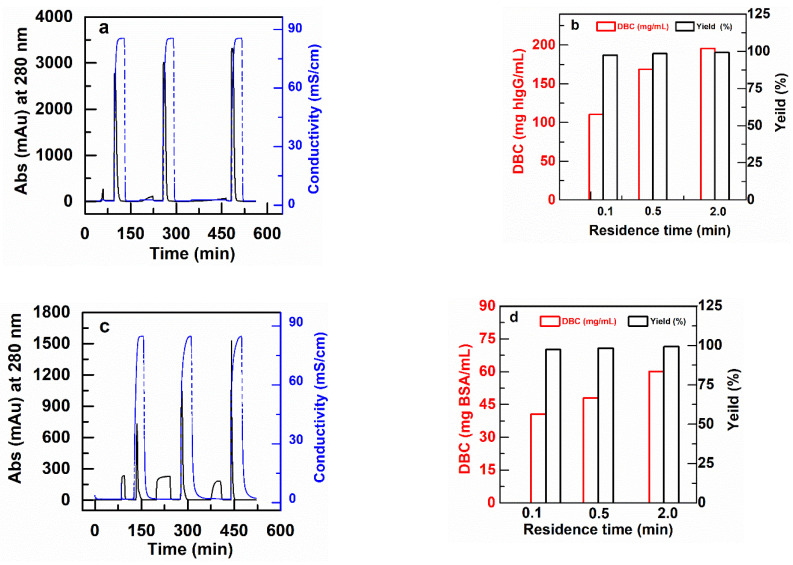
Typical chromatograms for DBC measurement of membrane packed columns (2.5 cm I.D) for RT of 0.1, 0.5 and 2.0 min. (**a**) PBT-GMA-SO_3_ membrane column (feed: 2 mg hIgG/mL) and (**b**) DBC for hIgG together with recovered yield (eluted vs. bound), (**c**) PBT-GMA-DEA membrane column (feed: 2 mg BSA/mL), and (**d**) DBC for BSA with recovered yield.

Figure 6 shows example chromatograms for some of these experiments at residence times of 0.1 min, 0.5 min and 2.0 min, together with the results for DBC and yields of bound protein that result. Note that the DBC values are significantly lower than the static equilibrium binding capacities described in the previous section. The reason for this is that with residence times of 2 min or less, the proteins do not have the time to diffuse through the grafted layer. Figure 6 also shows that the yields, or recovery of bound protein after elution, is nearly 100% in all cases.

Table 3 tabulates all of the measured DBC at all residence times and protein concentrations, as well as the pressure drops per unit length in the columns. The same number of layers of an AEX cellulose membrane show a lower DBC (approximately 30 mg BSA/mL) [41]. A commercial monolith functionalized by DEA exhibits a slightly larger biding capacity of 65 mg BSA/mL than that measured in this study at 2 min RT for the PBT AEX membrane. However, the PBT-CEX membranes shows a DBC for IgG capture that is more than twice the 53 mg hIgG/mL binding capacity found in a monolith with the same bed volume [38]. The DBC of monolithic disks were estimated from the plots of breakthrough curves reported by Hahn et al. [38] as the product of the feed concentration times the breakthrough volume divided by the bed volume (i.e., DBC = C_0_·V_BT_/V_BED_).

The results in Table 3 show that the dynamic binding capacities of the membranes for BSA and polyclonal hIgG, in general, do not vary much with feed concentrations in the range from 2 to 10 mg BSA/mL or hIgG/mL. However, for each experiment at a fixed protein concentration, there is a significant decrease in DBC as the residence time drops from 2.0 min to 0.1 min. This is a clear indication of diffusional limitations within the grafted layer at the shortest residence times that are not common in membranes and monoliths [9,38]. This effect is indeed compensated by the high binding capacity of these nonwoven membranes. The pressure drops per unit length for all the experiments were lower than 0.43 MPa/cm, even at the faster flow rates and shorter residence times.

There is an interesting effect at short residence times (0.1 min) where the DBC decreases as the product concentration in the feed increases from 2 to 10 mg/mL for both the anion and cation exchange membranes, the effect being stronger for anion exchangers. This effect is particularly pronounced for the DBC of the AEX membrane for binding BSA. At the lower concentrations there is much more volume of sample necessary to saturate the column than at the higher concentrations, so sample is being introduced for a longer time for the low concentrations than the high concentrations. It is possible that the longer time for these experiments results in additional time for proteins to diffuse into the grafted layers. Another possibility is that the DBC for the very high ligand density membranes is hindered by the close proximity of the charged groups. The effect is much more evident for the AEX membrane, which has the much larger ligand density as shown in Figure 5, and much less obvious for CEX membrane with its correspondingly lower ligand density. Evidence for this drop in DBC at increased ligand density has been identified by Vicente et al., [42] in membrane chromatography systems, and Hardin et al., in chromatography resins [43]. The developed ion-exchange nonwoven membranes are able to maintain more than 98% of their initial dynamic binding capacities in the cyclic test presented in Figure S3.

### 3.5. Purification

#### 3.5.1. Antibody Purification Using Cation Exchanger PBT-GMA-SO_3_

**Figure 7:**
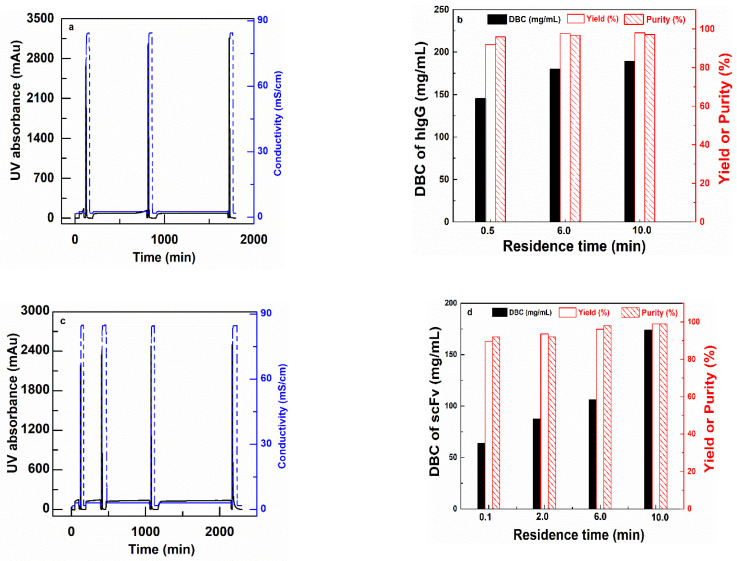
Bind and elute cycles of hIgG (**a**) and (**b**) and scFv (**c**) and (**d**) for PBT-GMA-SO_3_ membranes at different residence times. Chromatograms (**left**) and DBC100%, Yield and Purity (**right**) as a function of residence times.

CEX (PBT-GMA-SO_3_) nonwoven membranes were tested for purification of antibody (hIgG) and antibody fragments (scFv) spiked into clarified CHO cell culture supernatants. The purification was performed in separate sets of experiments, at different residence times (0.1, 6.0 and 10.0 min for hIgG purification and 0.1, 2, 6 and 10 min for scFv purification) by feeding 150 mL of samples containing 2 mg/mL of the target protein in solution. The chromatograms and the results in terms of hIgG DBC, yield, and purity are shown in Figure 7, while the SDS-PAGE analysis is reported in the Supplemental Information (Figure S4). The tabulated results for both experiments are shown in Table 4.

The membranes are able to capture both hIgG and scFv with very good selectivity from the impurities present in the CHO cell culture supernatant. The Protein G analysis of the chromatographic fractions for hIgG reveals that the purity of the antibody increased from 73.30% in the feed to 97.07% in the eluted samples with 98% yield with a 10 min RT as shown in Table 4. Further, the membrane demonstrated a DNA and HCP clearance with LRV of 2.22 and 1.55, respectively. These values implied that the membranes were able to clear 99% DNA and 95% HCP impurities from the feed samples in a single step. 

The PBT-GMA-SO_3_ membrane was also able to purify the smaller size antibody fragment (scFv) (27 kDa) from CHO cell culture supernatants. The collected elution fractions were directly loaded into a size-exclusion column (SEC) to estimate the purity of the recovered scFv. The purity of scFv increased from 66% in the feed to almost 99% in the eluted samples. The results indicated (Table 4) that at longer RT there is a complete recovery of scFv (about 99%) with a binding capacity of 174 mg scFv per mL of membrane. The HCP and DNA removal of the scFv samples were above 95% that is 1.81 and 1.51 LRV, respectively. At fast loading of 15 mL/min (0.1 min RT) still high values of about 90% yield and purity of scFv in the eluted samples were obtained as shown in Table 4. These suggest that there are some diffusion limitations, but even at short RT (0.1 min) the membranes have very good performances.

#### 3.5.2. PBT-GMA-DEA Membrane for DNA Removal

The PBT-GMA-DEA nonwoven membranes were tested in flow-through mode to remove DNA treated as an impurity in a mixture containing hIgG or scFv. Preliminary experiments were performed by injecting 20 µL pure CHO-DNA at a concentration of 1 µg/mL into the membrane column using 20 mM Tris-HCl, pH 7.0 as equilibration buffer. 95% of the feed DNA was recovered with a binding capacity of 12 µg DNA/mL membrane at a residence time of 2 min. After this positive result, tests with hIgG (or scFv) and DNA were performed.

**Figure 8:**
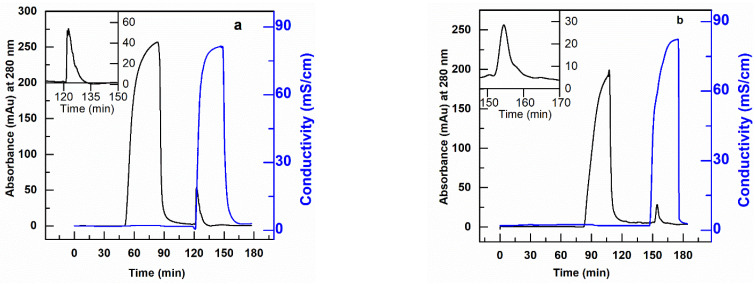
Removal of DNA from hIgG (**a**) and scFv (**b**) protein solution using anion-exchange PBT-GMA-DEA membrane operated in flow-through mode. The target proteins are collected in the flow through fraction and DNA in the elution peak. The insets show higher magnification of eluted DNA peak.

In particular, loads composed of 10 µg of CHO-DNA in 10 mL solutions of hIgG (or scFv) at a concentration of 2 mg/mL of protein in 20 mM Tris-HCl, pH 7.0 were fed to the AEX membrane column at various residence times and the DNA eluted in 1 M NaCl for quantification. The resulting chromatograms for the removal of DNA from hIgG and scFv solutions are shown in Figure 8 and tabulated in Table 5. The results in this table show that the PBT-GMA-DEA nonwoven membrane provides high levels of DNA clearance from hIgG without much loss of the protein. The SDS-PAGE analysis (Figure S5) demonstrate that the elution fractions are nearly free of the target protein. Further, the Picogreen analysis confirmed that the elution peaks as shown in Figure 8a,b corresponded to DNA.

The obtained results indicate that the membrane achieved more than 2.6 LRV DNA with a binding capacity of 3.44 µg/mL and 90% hIgG recovered in the flow-through. However, when using scFv solution the DNA clearance of the PBT-GMA-DEA membranes was more moderate, with a value around 1.0 LRV. Increasing the buffer pH to 8.0 did not show the any improvement, whereas a loading step at 1.83 cm/h (10 min RT) in 20 mM Tris-HCl, pH 7.0 achieved a higher improved dynamic capacity (4.1 µg DNA/mL) and 95% recovery of scFv in the flow-through. The studies revealed that the PBT-GMA-DEA membranes can be an excellent alternative to remove trace impurities in a polishing step.

## 4. Conclusions

Nonwoven PBT membranes with UV grafted GMA layers on the surface were modified by covalent attachment of sulfonate and secondary amines ligands to create cationic and anionic membranes for protein purification. The grafted PBT nonwoven material maintained most of its initial porosity after functionalization, resulting in membranes with very low pressure drops. SEM analyses indicated that the functionalized membranes had conformal and uniform GMA coatings around each fiber. Static equilibrium protein binding capacity measurements showed a direct and linear correlation between ligand density and the amount of protein adsorbed electrostatically to the surface of the fibers. The static binding studies also indicated that the highest binding capacities were obtained with grafted layers corresponding to 20% weight again after grafting. The binding equilibrium binding capacity for hIgG to the cationic membrane was as high as 700 mg hIgG/g membrane. The static, equilibrium anion exchange binding capacity for BSA was over 1300 mg BSA/g of membrane.

Dynamic binding capacities of the cationic membrane for hIgG of around 200 mg hIgG/mL were observed for residence times > 0.1 min. These binding capacities are similar to the binding capacities observed for the best cationic resins, but with much lower pressure drops and shorter residence times that are possible in chromatographic columns. The anion exchange membrane binding capacity for BSA was found to be around 55 mg BSA/mL of membrane in the same range of residence times. The cation exchange membranes were able to purify hIgG and scFv fragments from CHO cell supernatant with high yield and purity. The anion exchange membranes were able to remove high levels of DNA from mixtures of hIgG and scFv fragments with little loss of the products. Initial studies indicate that the membrane does not lose functionality with repeated cycles of use, but additional work on long-term stability needs to be done. Due to its excellent physical properties, high binding capacity and selectivity, low operating pressure drop at high linear velocities, and high productivity in a bind-and-elute mode, this type of membrane shows significant promise for single-use purification devices in biopharmaceutical manufacturing.

## Figures and Tables

**Table 1:** Porosity, basis weight and permeability of 20% weight gain nonwoven membranes.

	PBT Nonwoven	PBT-GMA	PBT-GMA-SO_3_	PBT-GMA-DEA
*Φ* (%)	78.48 ± 0.45	75.20 ± 0.47	74.19 ± 0.53	73.06 ± 0.67
Basis Weight (g/m^2^)	55.50 ± 0.26	65.58 ± 0.28	71.47 ± 0.21	75.29 ± 0.39
*κ* (m^2^)	3.28 × 10^−15^	3.15 × 10^−15^	2.69 × 10^−15^	1.82 × 10^−16^

**Table 2:** Static Binding Capacity (SBC) of unmodified PBT, and PBT-GMA grafted membranes.

Nonwoven Membrane	SBC (mg BSA/g Membrane)	SBC (mg hIgG/g Membrane)
PBT	9.68 ± 0.13	7.17 ± 0.86
PBT-GMA	8.22 ± 3.20	5.57 ± 0.19

**Table 3:** Comparison of the DBC of BSA (for AEX) or hIgG (for CEX) at different feed concentrations and RTs.

Membrane	Load BSA or hIgG c (mg /mL)	DBC (mg/mL)			ΔP/Bed Height (MPa/cm)
		RT 0.1 min	RT 0.5 min	RT 2 min	
^1^ PBT-GMA-SO_3_ (CEX-membranes)	2	106.20 ± 4.81	165.41 ± 2.05	199.08 ± 1.75	0.35
	4	118.11 ± 1.31	176.52 ± 0.98	199.64 ± 0.95	0.40
	6	116.60 ± 3.27	181.71 ± 1.53	200.28 ± 1.08	0.42
	8	98.31 ± 6.24	180.72 ± 1.03	199.01 ± 1.10	0.36
	10	91.38 ± 5.17	178.27 ± 2.48	193.56 ± 2.39	0.37
^2^ PBT-GMA-DEA (AEX-membranes)	2	40.62 ± 3.61	48.01 ± 1.11	60.19 ± 0.93	0.81
	4	37.69 ± 3.04	44.78 ± 1.63	58.20 ± 1.32	0.96
	6	36.51 ± 2.96	42.61 ± 1.84	54.62 ± 1.78	1.02
	8	35.06 ± 3.48	41.93 ± 2.47	52.20 ± 2.03	1.11
	10	34.19 ± 2.61	41.16 ± 3.05	49.39 ± 2.97	1.16

^1^ CEX-membranes packed column (0.442 g and 1.36 mL). ^2^ AEX-membranes (0.463 g and 1.57 mL).

**Table 4:** Performance of PBT-GMA-SO_3_ membranes for the purification of antibodies (hIgG and scFv) from CHO cell supernatant.

Protein	RT (min)	DBC (mg/mL)	^1^ Yield (%)	Purity (%)	HCP (LRV)	DNA (LRV)
hIgG	0.1	144.97	91.90	95.92	1.16	1.92
	6	180.03	97.54	96.68	1.26	2.18
	10	189.17	98.00	97.07	1.55	2.22
scFv	0.1	63.73	89.58	91.87	1.22	1.36
	2	87.32	93.55	92.07	1.52	1.39
	6	106.17	96.14	97.99	1.70	1.48
	10	174.00	98.91	98.93	1.81	1.51

^1^ Yield calculated as the ratio of eluted vs. bound.

**Table 5:** DNA clearance and protein recovery using nonwoven anion-exchange PBT-GMA-DEA membranes.

Protein	RT (min)	DBC (µg/mL)	Recovery hIgG/scFV (%)	DNA (LRV)
hIgG	5	3.44	90.05	2.61
^1^ scFv	2	2.04	90.30	0.95
	5	2.26	93.17	1.29
	10	4.06	94.87	1.23

^1^ Solution of 2 mg scFv/mL prepared in 20 mM Tris pH 8.0.

## Supplementary Materials

The following are available online at https://www.mdpi.com/2077-0375/11/3/181/s1, Figure S1: Ligand density at different reaction times, Figure S2: SBC of IEX membranes vs. storage time, Figure S3: DBC of IEX membranes over consecutive cycles, Figure S4: SDS-PAGE of collected fractions for purification of hIgG and scFv from CHO cell supernatant using PBT-GMA-SO_3_ membranes, Figure S5: SDS-PAGE of collected fractions for DNA removal from hIgG and scFv solutions using PBT-GMA-DEA membranes.
