# Supplementary material for: Nonwoven Ion-Exchange Membranes with High Protein Binding Capacity for Bioseparations
Source: Membranes (Basel). 2021 Mar 6;11(3):181. doi: 10.3390/membranes11030181 (PMC8001514; doi:10.3390/membranes11030181)
Supplement: Supplementary file 1 [file membranes-11-00181-s001.pdf]

membranes

MDPI

Supplementary Material

Nonwoven Ion-exchange Membranes with High Protein Binding Capacity for Bioseparations

Solomon Mengistu Lemma, Cristiana Boi and Ruben G. Carbonell

1. Ligand density optimization

The ligand density of the IEX membranes at different times of reactions as shown in Figure S-1. The CEX membrane achieved its optimal ligand density after 7 hours of reaction. On the contrary, the preparation of weak AEX required 16 hours to open the epoxy group rings and additional 16 h in 100 mM sulfuric acid to open the remaining unopen epoxy groups. The ligand densities used in the study were in the range of 0.35 to 0. 55 mmol/g and 1.10 to 1.40 mmol/g for cations and anion exchange membranes, respectively.

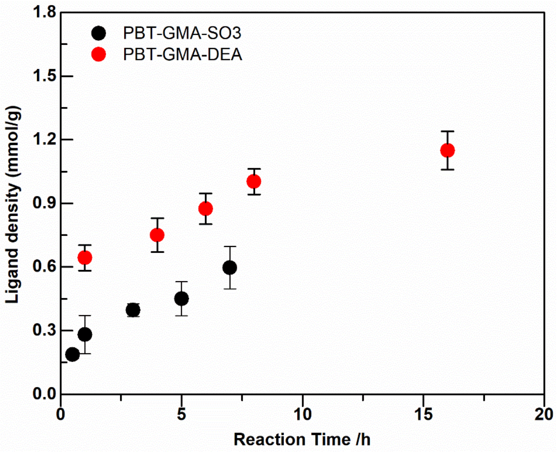

Figure S-1. Ligand density of cation, PBT-GMA-SO3, and anion, PBT-GMA-DEA, exchanger membranes.

2. Stability of ion exchange membranes on storage conditions

The static binding capacity was measured to evaluate the stability of the ligands on the IEX membranes over three months storage at 4 °C and at room temperature. The membrane are remained stable over 100 days of storage both at cold or room temperature conditions, as shown in Figure S-2.

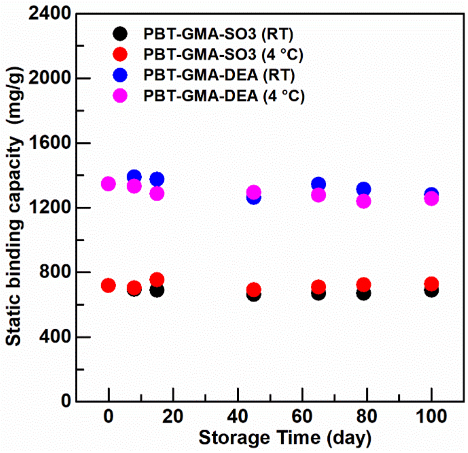

Figure S-2. Static binding capacity of ion-exchange membranes vs storage time at room temperature (RT) and 4 °C in air sealed containers.

3. Cycled dynamic binding capacity for repeated use

The measurement focused on bind-and-elute chromatographic operations were applied to investigate the reusability of the immobilized ligands on PBT nonwoven membranes over subsequint cycles. The stability of ligands in repeated experiments was tested using protein concentration of 2 mg/mL in the feed and a loading flow rate of 3 mL/min, corresponding to 0.5 min residence time.

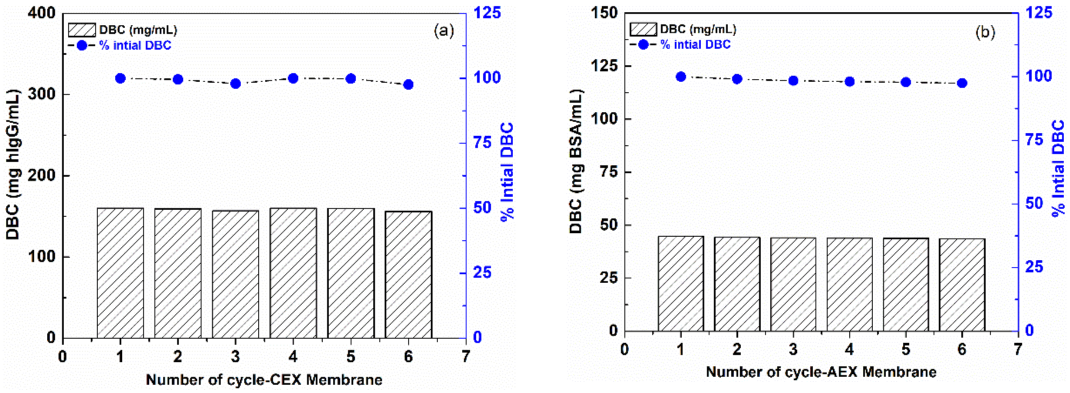

Figure S-3. DBC of PBT-nonwoven ion-exchange membranes over 6 complete bind and elute cycles. (**a**) cyclic test of hIgG in dynamic binding (158.68 ± 1.75 mg/mL), and (**b**) cyclic dynamic binding capacity of BSA (44.07 ± 0.42 mg/mL).

The cation exchange PBT membranes retained 99% of the initial hIgG dynamic binding capacity. In the same conditions, the anion exchange PBT membranes maintained about 98% of BSA binding. No substantial drop in the binding capacities was observed on the functionalized nonwovens during six cycles as shown in Figure S-3. Therefore, the developed ion-exchange nonwoven membranes are promising candidates for single-use disposable devices with high protein binding with stable ligands over multiple usages.

4. Selectivity of PBT functionalized membranes

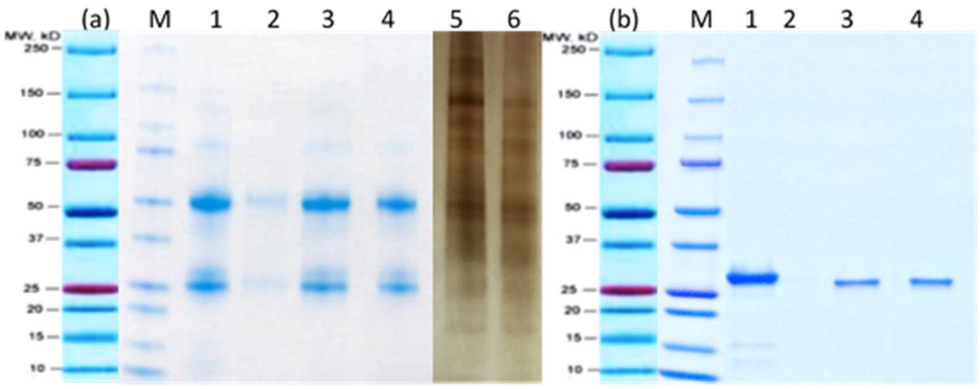

Figure S-4. Coomassie blue-stained SDS-PAGE with different fractions from cation exchanger PBT-GMA-SO_3 columns, which were used for purification of hIgG and scFv from CHO cell supernatant: in both (a) and (b): Lane (M) Bio-Rad SDS-molecular weight marker; Lane (1) fed of hIgG/scFV with CHO cell supernatant; Lane (2) flow-through; Lane (3) eluted hIgG/scFV; (4) standard hIgG or scFV. Silver-stained SDS-PAGE of unpurified CHO cell supernatant as received (Lane 5) and diafiltered with 3KD TFF to reduce the impurities (Lane 6).

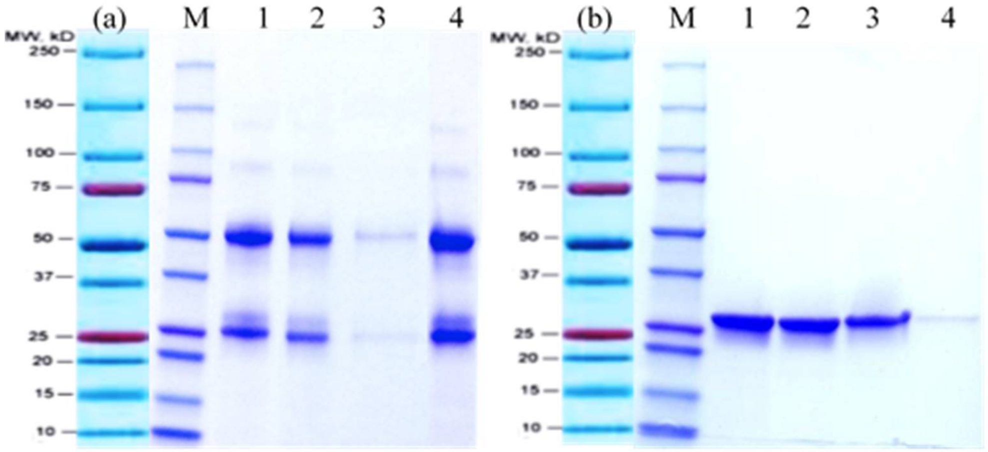

Figure S-5. SDS-PAGE of the removal of DNA from hIgG/scFv protein solution using anion-exchange PBT-GMA-DEA membrane. (a) DNA concentrate spiked in hIgG solution: lane M, molecular weight markers; lane 1, fed (hIgG + DNA); lane 2, Flow-through; lane 3, elution and lane 4, standard hIgG. (b) DNA concentrate spiked in scFv solution: lane M, molecular weight markers; lane 1, pure standard scFv; lane 2, fed (scFv + DNA); lane 3, flow-through and lane 4, elution of the sample.
